# Fatal Postoperative Pulmonary Tumor Thrombotic Microangiopathy: A Rare and Fulminant Complication of Large Cell Lung Carcinoma

**DOI:** 10.7759/cureus.93892

**Published:** 2025-10-05

**Authors:** Yuri Hiramatsu, Mizuki Muranaka, Shouta Sogabe, Yoshihiro Ohishi, Kazunori Tobino

**Affiliations:** 1 Respiratory Medicine, Iizuka Hospital, Fukuoka, JPN; 2 Pathology, Iizuka Hospital, Fukuoka, JPN

**Keywords:** autopsy, disseminated intravascular coagulation (dic), large cell lung carcinoma, paraneoplastic syndrome, pulmonary hypertension, pulmonary tumor thrombotic microangiopathy

## Abstract

Pulmonary tumor thrombotic microangiopathy (PTTM) is a paraneoplastic pulmonary vascular process characterized by microscopic tumor emboli with fibrocellular intimal proliferation, causing rapid progressive pulmonary hypertension and right heart failure. We report a 72-year-old male with stage IIB large cell lung carcinoma who developed fulminant respiratory failure two months after curative-intent lobectomy, just prior to adjuvant chemotherapy. On presentation, he had multiorgan failure and disseminated intravascular coagulation with a markedly elevated D-dimer. Despite intensive supportive care, he died within hours. Autopsy confirmed PTTM with widespread tumor emboli occluding small pulmonary arteries and arterioles, together with previously unrecognized systemic metastases. This case illustrates that PTTM may precipitate catastrophic decompensation even soon after apparently curative surgery and supports maintaining a high index of suspicion for this syndrome in large cell lung carcinoma patients presenting with acute cardiorespiratory failure.

## Introduction

Pulmonary tumor thrombotic microangiopathy (PTTM) is a rare but highly lethal paraneoplastic syndrome characterized by widespread embolization of microscopic tumor cells into the pulmonary microvasculature. This initial event triggers a local coagulation cascade and subsequent fibrocellular intimal proliferation, which leads to progressive vascular stenosis, severe pulmonary hypertension, and ultimately, acute right heart failure. Although PTTM has been linked to various malignancies, gastric adenocarcinoma is by far the most common primary cancer, responsible for approximately 59% of reported instances, with breast and lung cancers also being notable causes [1]. The clinical diagnosis of PTTM before death is notoriously difficult due to its fulminant progression and nonspecific symptoms, resulting in the majority of diagnoses being made only at autopsy [2].

While lung cancer is a recognized origin for PTTM, the existing literature predominantly links this condition to the adenocarcinoma subtype. In contrast, large cell lung carcinoma (LCLC) is a less frequent and poorly differentiated subtype of non-small cell lung cancer (NSCLC), noted for its aggressive clinical behavior and generally poor prognosis [3]. The development of PTTM as a direct complication of LCLC, however, is an event that has been infrequently documented, representing a significant gap in our current understanding [1,2,4]. The motivation for presenting this case is therefore to highlight this rare association, expanding the spectrum of malignancies known to precipitate PTTM and alerting clinicians to a potentially under-recognized fatal risk in patients with this specific histology.

We report a fatal case of PTTM presenting two months after a LCLC resection. This case highlights the clinical and pathological features of this rare condition and the need for clinicians to consider PTTM even after recent curative treatment.

## Case presentation

**Figure 1:**
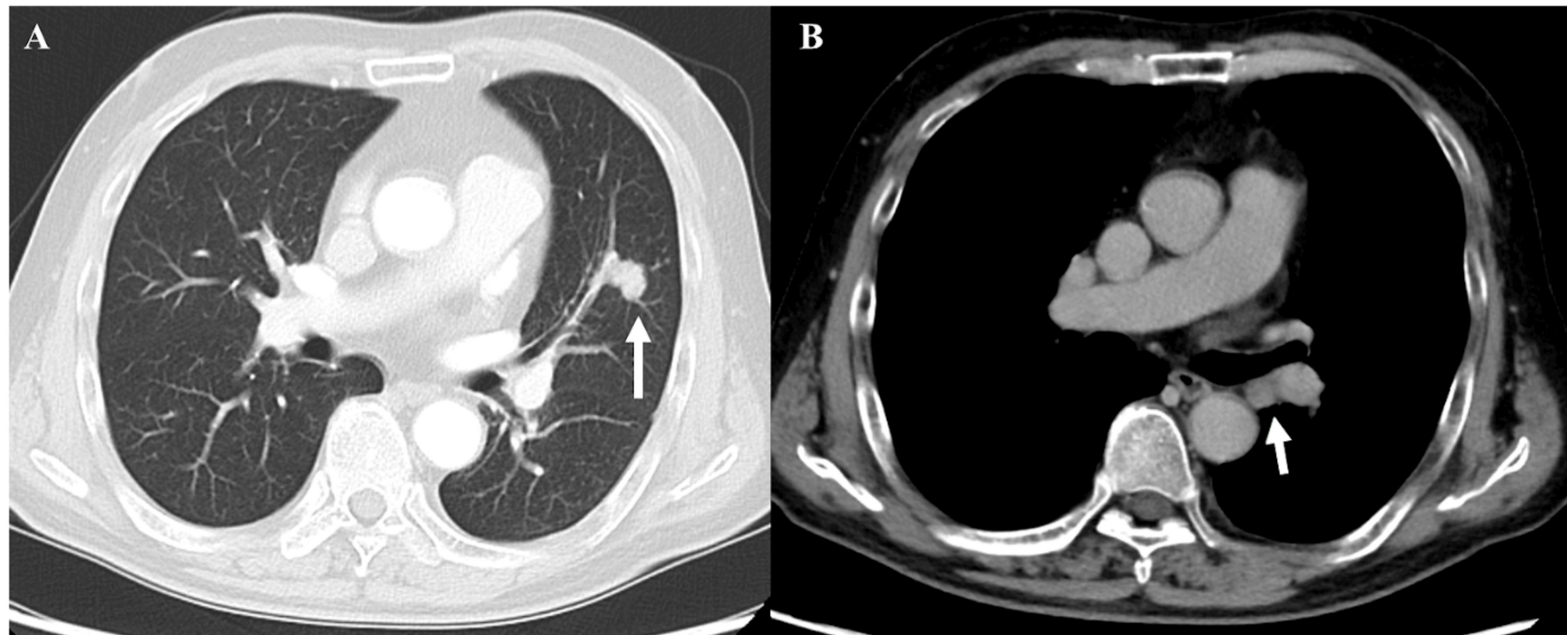
Preoperative CT findings. Axial view of the chest CT scan performed prior to surgery, revealing a nodule in the left upper lobe (A, indicated by the white arrow) and evidence of mediastinal lymph node metastasis (B, also indicated by the white arrow in the mediastinal window).

A 72-year-old male with a 50-pack-year smoking history presented to the emergency department with acute respiratory distress. He had quit smoking two years earlier. His medical history was notable for hypertension, dyslipidemia, and a recent diagnosis of LCLC based on preoperative imaging (Figure 1).

Approximately two months prior to this presentation, he had undergone a thoracoscopic left upper lobectomy and lymph node dissection for this malignancy, with pathology confirming stage IIB (pT1bN1M0) disease. Lymph nodes from nine stations (#4L, #5, #6, #7, #10, #11, #12u, #12L, and #13) were dissected. In total, 30 nodes were evaluated, nine of which were positive for metastasis (#6, 2/4; #10, 2/3; #12u, 2/2; #13, 3/10). Immunohistochemical staining showed the following profile: TTF-1 (−), napsin A (−), p40 (−), CK14 (−), CD56 (+), chromogranin A (−), synaptophysin (focally +), and MIB-1 labeling index of 70%. The patient presented for evaluation just before his first scheduled cycle of adjuvant chemotherapy. Meanwhile, the patient had reported back pain and chest pain beginning approximately 10 days prior to the acute event. There had been no complaints of respiratory or airway symptoms. Preoperative PET-CT demonstrated intense fluorodeoxyglucose (FDG) uptake in the known left upper lobe S4 mass and in an enlarged left hilar lymph node, without other abnormal foci. Serum pro-gastrin-releasing peptide (proGRP) was mildly elevated at 95.6 pg/mL 10 days before the acute event and at 85.4 pg/mL immediately prior to surgery. Circulating tumor cell analysis was not performed.

On arrival, the patient was in severe respiratory distress with a markedly altered level of consciousness (Japan Coma Scale III-300). His respiratory rate was 30 breaths per minute, and his oxygen saturation was 95% while receiving 10 L/min of oxygen via a non-rebreather mask. Physical examination was significant for icteric sclerae and bilaterally diminished breath sounds. Due to his inability to maintain adequate oxygenation and protect his airway, he was immediately intubated for mechanical ventilation and admitted to the intensive care unit (ICU).

Initial laboratory investigations (Table 1) revealed a crisis of coagulopathy and multi-organ failure. There was profound evidence of disseminated intravascular coagulation (DIC), characterized by a massively elevated D-dimer (70.6 μg/mL), a prolonged prothrombin time-international normalized ratio (PT-INR, 2.39), and a prolonged activated partial thromboplastin time (42.4 seconds). This occurred in the presence of mild thrombocytopenia (platelet count, 12.7×10⁴/μL) and anemia (hemoglobin, 10.9 g/dL). Inflammatory markers were strikingly high, with a lymphocyte-predominant leukocytosis (white blood cell count, 17,910/μL) and a C-reactive protein of 16.39 mg/dL. The results confirmed severe multi-organ dysfunction, including acute liver injury (lactate dehydrogenase, 12,690 U/L) and renal failure (creatinine, 1.8 mg/dL) with hyperkalemia (5.8 mEq/L). Arterial blood gas analysis demonstrated severe metabolic acidosis (pH, 7.193) driven by profound lactic acidosis (blood lactate, 93.1 mg/dL).

**Table 1:** Results of laboratory tests. PaCO2: partial pressure of carbon dioxide; PaO2: partial pressure of oxygen; HCO3: bicarbonate.

Test	Results	Reference range
White blood cells	17910/μL	3300-8600/μL
Neutrophils	42.7%	39.5-74.5%
Lymphocytes	45.6%	20.9-54.1%
Monocytes	5.7%	3.6-9.8%
Eosinophils	0.8%	0.0-8.1%
Red blood cells	3.6×10^6/μL	4.35-5.55×10^6/μL
Hemoglobin	10.9 g/dL	13.7-16.8 g/dL
Hematocrit	32.90%	40.7-50.1%
Mean corpuscular volume	90.3 fL	83.6-98.2 fL
Platelet	12.7×10^4/μL	15.8-34.8×10^4/μL
Aspartate aminotransferase	406 U/L	13-30 U/L
Alanine aminotransferase	88 U/L	10-42 U/L
Lactate dehydrogenase	12690 U/L	124-222 U/L
Alkaline phosphatase	489 U/L	38-113 U/L
γ-glutamyl transpeptidase	750 U/L	13-64 U/L
Creatine kinase	633 U/L	28-248 U/L
Total bile acid	3.5 mg/dL	0.4-1.5 mg/dL
Albumin	3.9 g/dL	4.1-5.1 g/dL
Urea nitrogen	71 mg/dL	8-20 mg/dL
Creatinine	1.8 mg/dL	0.65-1.07 mg/dL
Sodium	135 mEq/L	138-145 mEq/L
Potassium	5.8 mEq/L	3.6-4.8 mEq/L
Chloride	99 mEq/L	101-108 mEq/L
Calcium	11.0 mg/dL	8.8-10.1 mg/dL
Inorganic phosphate	6.5 mg/dL	2.7-4.6 mg/dL
Glucose	308 mg/dL	73-109 mg/dL
C-reactive protein	16.39 mg/dL	0-0.14 mg/dL
Prothrombin time (%)	28.5%	80-120%
Prothrombin time-international normalized ratio	2.39%	0.90-1.20%
Activated partial thromboplastin time	42.4 sec	24-32 sec
D-dimer	70.6 μg/mL	<1.0 μg/ml
pH (RM 10 L/min)	7.193	7.35-7.45
PaCO2 (RM 10 L/min)	34.2 Torr	36-45 Torr
PaO2 (RM 10 L/min)	84.8 Torr	86-107 Torr
HCO3- (RM 10 L/min)	12.9 Tprr	23-28 Torr
Base excess (RM 10 L/min)	-14.2 mmol/L	-2.3 - +2.3 mmol/l
Blood lactate	93.1 mg	4-16 mg/dl

**Figure 2:**
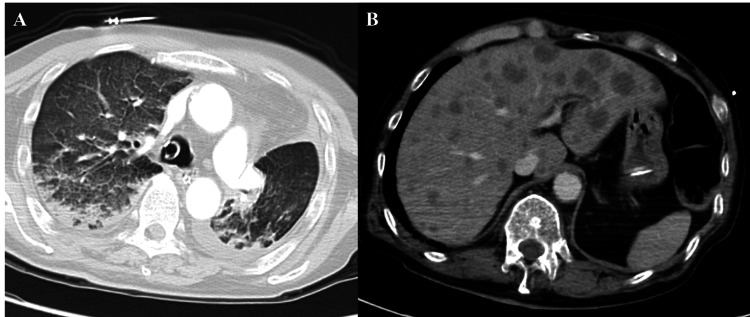
CT findings on admission. (A) An axial chest CT image shows diffuse ground-glass opacities and marked interlobular septal thickening. (B) An axial CT image of the upper abdomen reveals multiple, widespread hypodense lesions throughout the liver, consistent with metastatic disease.

A chest radiograph showed decreased permeability in both lungs, and a subsequent computed tomography (CT) scan demonstrated interlobular septal thickening, diffuse ground-glass opacities, bilateral pleural effusions, and multiple new hypodense lesions in the liver, consistent with metastatic disease. The main pulmonary artery measured approximately 360 Hounsfield units on contrast-enhanced CT, indicating that the timing was adequate to detect even subsegmental intraluminal filling defects. No central or segmental intraluminal filling defects were identified. Microvascular diseases, such as PTTM, may not be detectable on routine CT angiography. However, no findings suggestive of tumor embolization were observed (Figure 2). Nonetheless, the elevated pulmonary artery to aorta (PA:Ao) ratio indicates increased pulmonary vascular resistance, supporting a pathophysiologic scenario of PTTM-driven pre-capillary pulmonary hypertension culminating in acute right-sided failure.

An arterial pressure line was placed via the right radial artery in the emergency department. Continuous infusion of norepinephrine was initiated, and blood pressure stability was confirmed through the arterial line. As bradycardia developed, diluted epinephrine (1:10) and sodium bicarbonate were administered. Vascular access was subsequently established via the left internal jugular vein, after which the arterial line waveform disappeared. Further attempts to secure arterial access from the posterior tibial arteries, the left brachial artery, and other sites were unsuccessful. Bradycardia was attributed to worsening hyperkalemia and acidosis. Following admission to the ICU, continuous renal replacement therapy (CRRT) was promptly initiated at 5000 mL/hr, together with intravenous calcium, bicarbonate, and gastrointestinal treatment. An arterial pressure line was subsequently placed via the left femoral artery. Shortly thereafter, the heart rate abruptly decreased to approximately 30 beats per minute and progressed to asystole within about 10 seconds, necessitating the initiation of cardiopulmonary resuscitation (CPR). Despite approximately 30 minutes of resuscitative efforts, recovery was not achieved. After discussion with the patient’s family regarding the futility of further intervention, CPR was discontinued.

**Figure 3:**
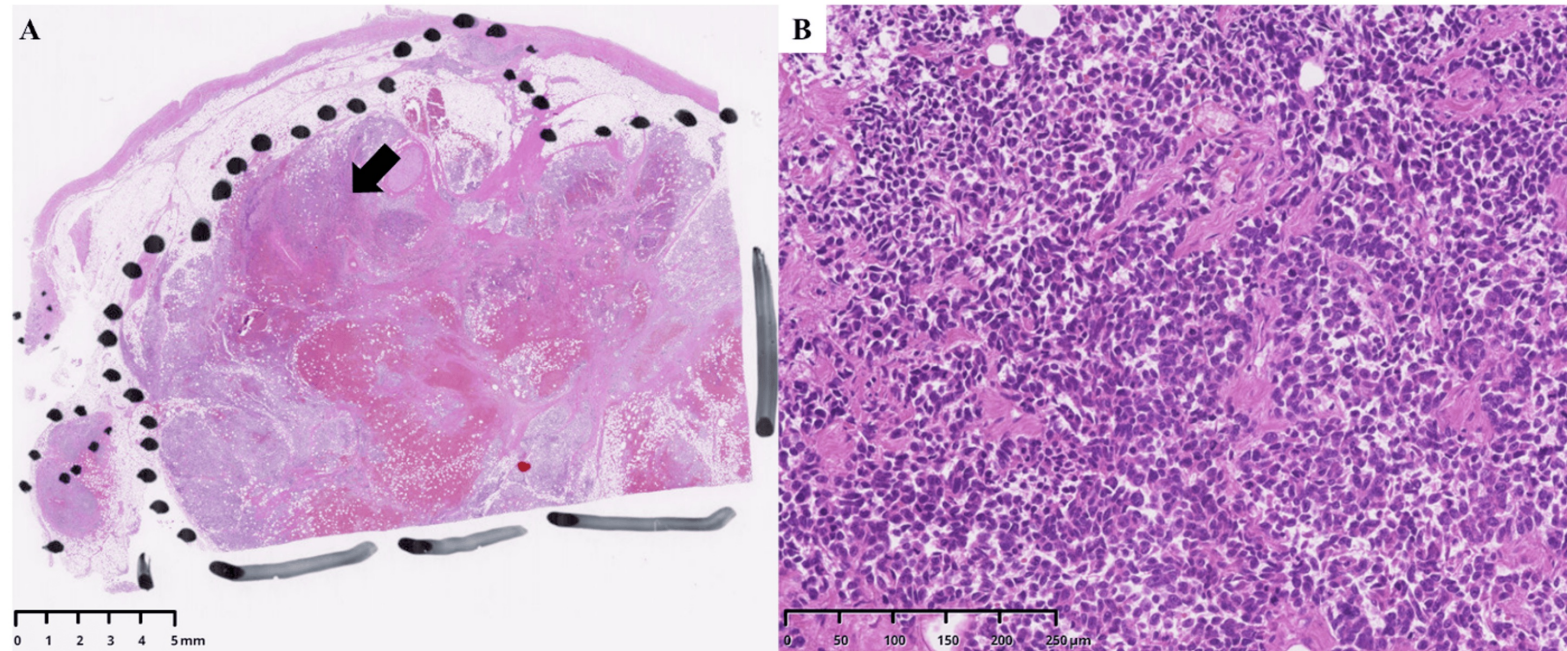
Histopathological findings of mediastinal lymph node metastasis. (A) Low-power view (hematoxylin and eosin stain) shows a mediastinal lymph node almost completely effaced by metastatic carcinoma. The dotted line demarcates the lymph node capsule, and the arrow indicates the area shown at higher magnification in panel B. (B) High-power view reveals sheets of poorly differentiated malignant cells with large pleomorphic nuclei, irregular nuclear contours, and prominent nucleoli, characteristic of large cell carcinoma.

Given the unclear etiology of the patient's rapid clinical deterioration, a pathological autopsy was performed with the family's consent. The examination revealed extensive mediastinal lymph node metastases (Figure 3).

**Figure 4:**
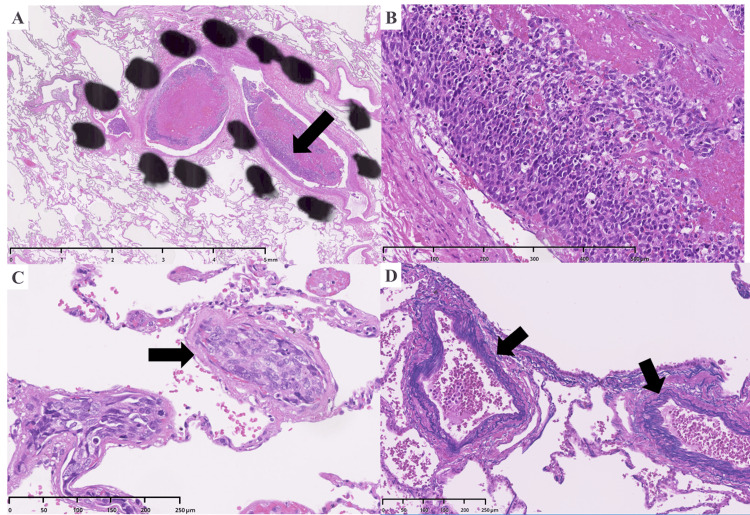
Histopathological findings of pulmonary tumor thrombotic microangiopathy (PTTM). (A) Low-power view (hematoxylin and eosin stain) shows a pulmonary artery occluded by a large tumor embolus. The arrow indicates the area magnified in panel B. (B) High-power magnification of the tumor embolus, composed of sheets of malignant cells. (C) A peripheral small vessel is shown to be completely occluded by tumor cells (arrow). (D) Verhoeff-Van Gieson (VVG) stain of another vessel highlights the characteristic fibrocellular intimal thickening causing stenosis of the vascular lumen (arrows).

Histological analysis of the lungs provided the definitive diagnosis: numerous tumor cell emboli and associated fibrin thrombi were identified within the small pulmonary arteries and arterioles, accompanied by fibrous intimal thickening (Figure 4).

**Figure 5:**
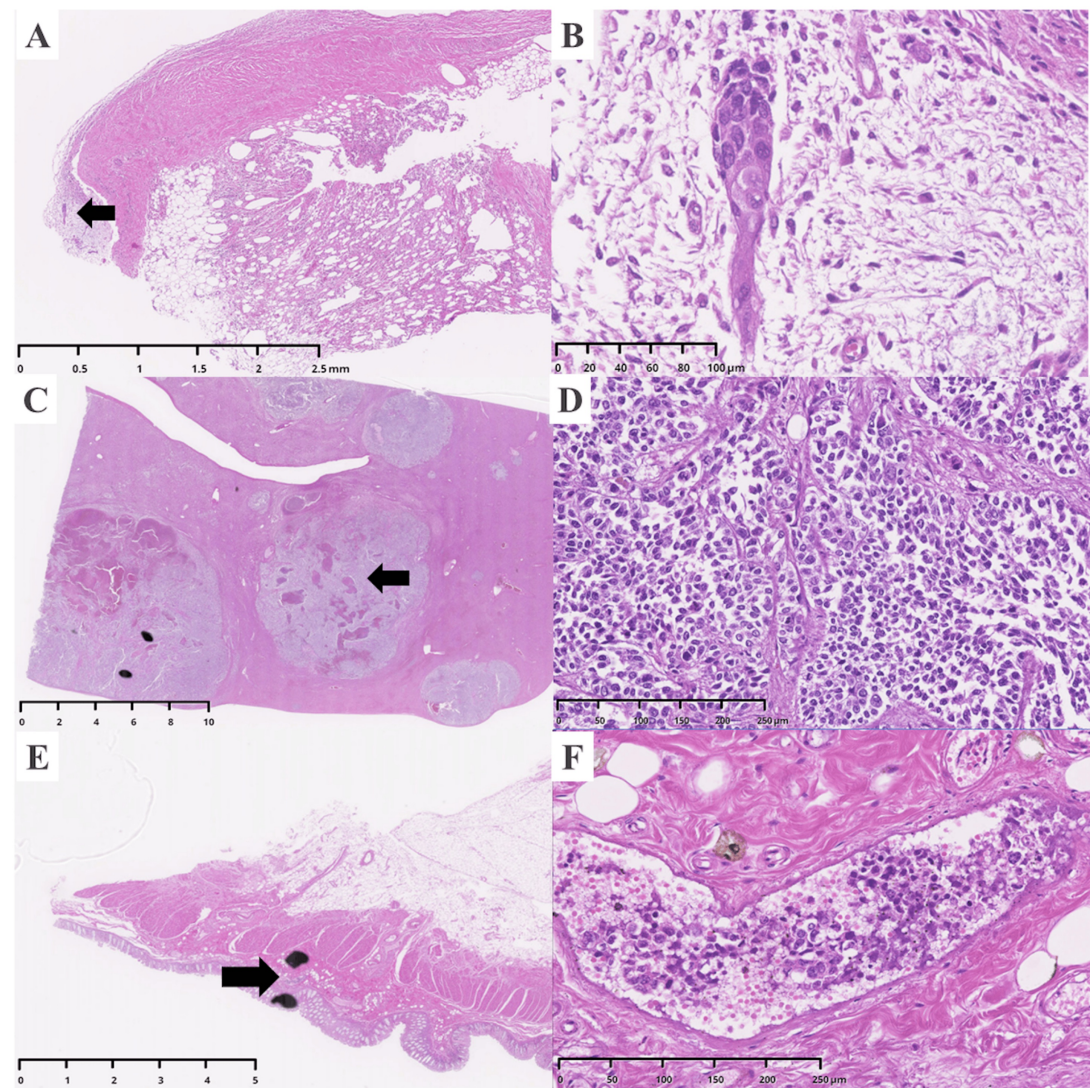
Widespread metastatic disease of large cell carcinoma was found at autopsy. Histological images (hematoxylin and eosin stain) confirm metastatic deposits in the pleura, liver, and colon. (A) Low-power view of a metastatic nodule in the pleura. The arrow indicates the area magnified in panel B. (B) High-power view showing infiltration of the pleura by malignant cells. (C) Low-power view of the liver showing a large, circumscribed metastatic nodule. The arrow indicates the area magnified in panel D. (D) High-power view demonstrating sheets of tumor cells replacing the normal hepatic parenchyma. (E) Low-power view of the colon wall showing a metastatic deposit. The arrow indicates the area magnified in panel F. (F) High-power view confirming tumor infiltration into the bowel wall.

These findings were pathognomonic for PTTM. The autopsy also confirmed the extensive nature of the malignancy, with metastatic deposits identified in the pleura, liver, and colon (Figure 5).

## Discussion

This report details a case of fatal PTTM that developed with extreme rapidity following the surgical resection of LCLC. The significance of this case is underscored by two primary findings. First, it establishes a rare link between PTTM and LCLC, a histological subtype infrequently implicated in this condition, which is most commonly associated with adenocarcinomas. Second, it highlights the exceptionally fulminant nature of the disease's progression in the immediate postoperative period, which poses a profound diagnostic challenge and illustrates the aggressive underlying biology of the malignancy despite recent curative-intent treatment. These findings contribute to a broader understanding of PTTM's potential etiologies and clinical presentations.

Our first key finding, the manifestation of PTTM in a patient with LCLC, is significant, given the established epidemiology of this syndrome. Whereas prior literature has overwhelmingly linked PTTM to adenocarcinoma, our case expands the clinicopathological spectrum by illustrating that LCLC can also drive this fatal entity. The literature overwhelmingly identifies adenocarcinoma as the causative histology in the majority of PTTM cases, with some autopsy series reporting this figure to be as high as 90% [4]. In stark contrast, a direct link with LCLC is scarcely reported. This apparent rarity, however, may be partially explained by a diagnostic artifact rooted in histological ambiguity. Given PTTM's strong association with adenocarcinomas, it is conceivable that poorly differentiated tumors causing this syndrome are preferentially classified as "solid adenocarcinoma," leading to an underestimation of LCLC's true incidence. More importantly, biological studies of LCLC provide a strong mechanistic rationale for this association. Despite sometimes showing lower microvascular density than other NSCLC subtypes, LCLC has been observed to exhibit a potent invasive phenotype, uniquely characterized by "vascular-like structures containing numerous tumor emboli" [5]. This observation provides direct evidence of LCLC's intrinsic capacity for aggressive vascular invasion and embolization, the critical initiating event of PTTM. Thus, our case is not merely a report of a rare co-occurrence but highlights a specific biological behavior of LCLC that makes it a plausible, albeit under-recognized, driver of this fatal condition.

Our second finding, the exceptionally fulminant postoperative deterioration, highlights the devastating clinical trajectory typical of PTTM and the diagnostic quagmire it creates. The patient's rapid progression from apparent stability to death within hours is consistent with the aggressive nature of this syndrome, for which the median survival after symptom onset is often measured in mere weeks [1]. The non-specific presentation of acute dyspnea is frequently misattributed to more common conditions like pneumonia or pulmonary thromboembolism, causing critical diagnostic delays [6]. What makes our case particularly instructive is its occurrence following a recent curative-intent lobectomy. This timeline suggests that despite the removal of the primary tumor and macroscopic nodal disease, aggressive and widespread micrometastases were already systemically established. The catastrophic right heart failure that ensued is a direct consequence of a rapid increase in pulmonary vascular resistance, driven by widespread microvascular occlusion from tumor emboli and subsequent fibrocellular proliferation [7]. This case, therefore, serves as a sobering illustration that PTTM reflects a state of disseminated malignancy that can override conventional staging paradigms, demonstrating that even after "complete" surgical resection, the inherent biological aggressiveness of cancers like LCLC can precipitate a fatal outcome.

The interpretation of this case carries significant clinical and research implications. Primarily, it mandates a high index of suspicion for PTTM in any patient with LCLC presenting with acute, unexplained cardiorespiratory failure, even following supposedly curative surgery. Given that invasive procedures like lung biopsy are often contraindicated in these hemodynamically unstable patients [7], a paradigm shift toward a rapid, multi-modal, and less invasive diagnostic strategy is warranted. This approach could combine findings from FDG-PET/CT, which may reveal diffuse pulmonary uptake, with right heart catheterization to confirm severe pre-capillary pulmonary hypertension and, crucially, to obtain a pulmonary artery blood sample for cytological analysis [7]. Achieving a rapid presumptive diagnosis through this "triad" is critical because it opens a window for immediate therapeutic intervention. This may include not only systemic chemotherapy for the LCLC but also targeted agents like imatinib or bevacizumab aimed at disrupting the underlying pathophysiology [8,9]. Furthermore, this case impels future research into exploring liquid biopsies for circulating biomarkers like osteopontin for the early detection of high-risk micrometastatic disease in these patients [10].

A targeted PubMed search (terms: “pulmonary tumor thrombotic microangiopathy” AND “large cell lung carcinoma”; last accessed: October 3, 2025) identified no prior reports directly associating PTTM with LCLC. While absence of evidence is not evidence of absence, this suggests considerable rarity. Only 5-mm CT slices were available; 1-mm high-resolution images were not obtained, which may have limited the detection of subtle interstitial or small-vessel changes.

## Conclusions

This case report describes the fatal manifestation of PTTM as a rare complication of LCLC, a malignancy with a demonstrated biological capacity for aggressive vascular invasion. The fulminant postoperative course highlights that PTTM can represent a state of disseminated disease not captured by conventional staging, rendering even recent "curative" surgery ineffective against its progression. The primary clinical lesson learned is the absolute necessity for a high index of suspicion for PTTM in any LCLC patient with acute cardiorespiratory decline. By adopting a rapid, multi-modal diagnostic approach, clinicians may be better positioned to identify this elusive syndrome and initiate potentially life-altering therapies. Ultimately, this case underscores the aggressive nature of LCLC and reinforces the need for vigilance against its most catastrophic complications.
